# Ebola preparedness in Sierra Leone during the 2026 Bundibugyo virus emergency: institutional findings and introduction of the Architectural and Operational Readiness Gap (AORG) framework

**DOI:** 10.11604/pamj.2026.54.39.53501

**Published:** 2026-06-09

**Authors:** Eric Nzirakaindi Ikoona, Lucy Namulemo, Mary Magdalene Sinnah, Mohamed Alex Vandi, Foday Sahr

**Affiliations:** 1 National Public Health Agency, 42A Main Motor Road, Wilberforce, Freetown, Sierra Leone,; 2 Foothills Community-Based Interventions, Kentucky, United States of America

**Keywords:** Ebola disease, Bundibugyo virus, filovirus, preparedness, mixed methods, Sierra Leone

## Abstract

**Introduction:**

the World Health Organization (WHO) declared the Bundibugyo Ebola virus disease (EVD) outbreak in the Democratic Republic of the Congo (DRC) and Uganda a Public Health Emergency of International Concern (PHEIC) on May 17, 2026; no licensed vaccine or specific therapeutic exists. Rapid preparedness assessments tend to capture plans more reliably than operational functionality. We propose the Architectural and Operational Readiness Gap (AORG) framework as an exploratory analytic lens, applied here in Sierra Leone.

**Methods:**

cross-sectional, convergent parallel mixed-methods design. In May 2026, the Emergency Preparedness and Response team at the National Public Health Agency of Sierra Leone, led by the manager, completed the Economic Community of West African States (ECOWAS) rapid assessment questionnaire. Two reviewers classified the 35 binary indicators as architectural or operational. Domain AORG was the percentage-point difference between scores. Open-text responses were analysed using rapid qualitative content analysis. Reporting follows the STROBE cross-sectional checklist, with GRAMMS and SRQR additionally applied to the mixed-methods and qualitative elements.

**Results:**

twelve of 15 architectural (80%) and 10 of 20 operational (50%) indicators were reported as in place; exploratory national AORG +30 percentage points. Largest positive AORGs: infection prevention and control (+100 pp; 0/5) and points of entry (+100 pp; 0/1). Risk communication showed inverse AORG (-67 pp). National laboratory capacity covered only Zaire ebolavirus. Four qualitative themes emerged: training; supplies and supply-chain fragility; non-functional infrastructure; decentralised local-language preparedness.

**Conclusion:**

Sierra Leone reported substantial preparedness architecture but a notable operational gap, concentrated in Bundibugyo diagnostics, infection prevention, isolation infrastructure and operationalisation of an existing emergency financing trust fund. Findings reflect one institutional position, not an independent audit. The AORG framework may support rapid mixed-methods preparedness measurement.

## Introduction

The World Health Organization (WHO) declared the Bundibugyo Ebola virus disease (EVD) outbreak in the Democratic Republic of the Congo (DRC) and Uganda a Public Health Emergency of International Concern (PHEIC) on May 17, 2026 [1,2]. The determination was made under Article 12 of the amended International Health Regulations (IHR) and preceded the IHR Emergency Committee, the first such occasion in IHR history [3]. The event did not meet the criteria for a pandemic emergency, the higher classification introduced through the 2024 IHR amendments that entered into force in September 2025 [4]. As of 20 May 2026, WHO reported 51 laboratory-confirmed cases of Bundibugyo virus disease (BVD) in the DRC across Ituri and North Kivu provinces, including Bunia and Goma. Uganda reported two laboratory-confirmed cases in Kampala, including one death. WHO also reported almost 600 suspected cases and 139 suspected deaths [2,3]. Bundibugyo virus is one of six recognised orthoebolaviruses. The International Committee on Taxonomy of Viruses renamed the genus Ebolavirus to Orthoebolavirus in April 2023 [5]. Two BVD outbreaks have been previously documented: Uganda in 2007 and DRC in 2012. Past case-fatality rates ranged from 30 to 50 percent [6,7]. No licensed vaccine or specific therapeutic exists for Bundibugyo virus [1,2].

Sierra Leone does not border either affected country. Three considerations make the country's preparedness position relevant. First, Sierra Leone has the most extensive recent national experience of Ebola disease control in West Africa: 14,124 cases and 3,956 deaths during the 2014 to 2016 epidemic [8,9]. Second, the country is an Economic Community of West African States (ECOWAS) member. Regional health security obligations run through the Regional Centre for Surveillance and Disease Control (RCSDC) and the West African Health Organization (WAHO). Third, African air connectivity and population mobility create defined onward transmission pathways. After the 2014 to 2016 epidemic, Sierra Leone enacted the Public Health Act, 2022 (Act No. 17 of 2023). The National Public Health Agency (NPHA) was launched on 14 December 2023, alongside the Public Health Emergency Trust Fund (PHETF) [10-12]. Preparedness measurement under the IHR uses three main instruments: annual States Parties self-assessment, periodic Joint External Evaluation, and the Universal Health and Preparedness Review [13-15]. The WHO Pandemic Agreement, adopted by the 78^th^ World Health Assembly on 20^th^ May 2025, is the most recent global addition. Its Pathogen Access and Benefit-Sharing annex remains under negotiation [16,17]. These tools share a recurring weakness. Self-reported and rapid-completion instruments capture formal establishment more reliably than current functionality. The distinction matters most in the first hours and days of a public health emergency.

We propose the Architectural and Operational Readiness Gap (AORG) framework as an exploratory analytic lens for rapid preparedness assessment, distinguishing architectural readiness (capacity on paper) from operational readiness (capacity in practice). Domain-level AORG = % architectural items reported in place - % operational items reported in place. The framework is not a validated preparedness index and depends on the underlying instrument, the accuracy of self-report, indicator set, item classification and the absence of external verification (Figure 1). The framework separates two dimensions. Architectural readiness is capacity on paper: plans, policies, designated facilities, signed agreements and documented mandates. Operational readiness is capacity in practice: trained staff in post, stocked supplies, equipped facilities, active financing and embedded routines. AORG is the percentage-point gap between the two. We applied AORG to a rapid mixed-methods institutional self-assessment completed by the NPHA in May 2026 using the RCSDC instrument.

**Figure 1:**
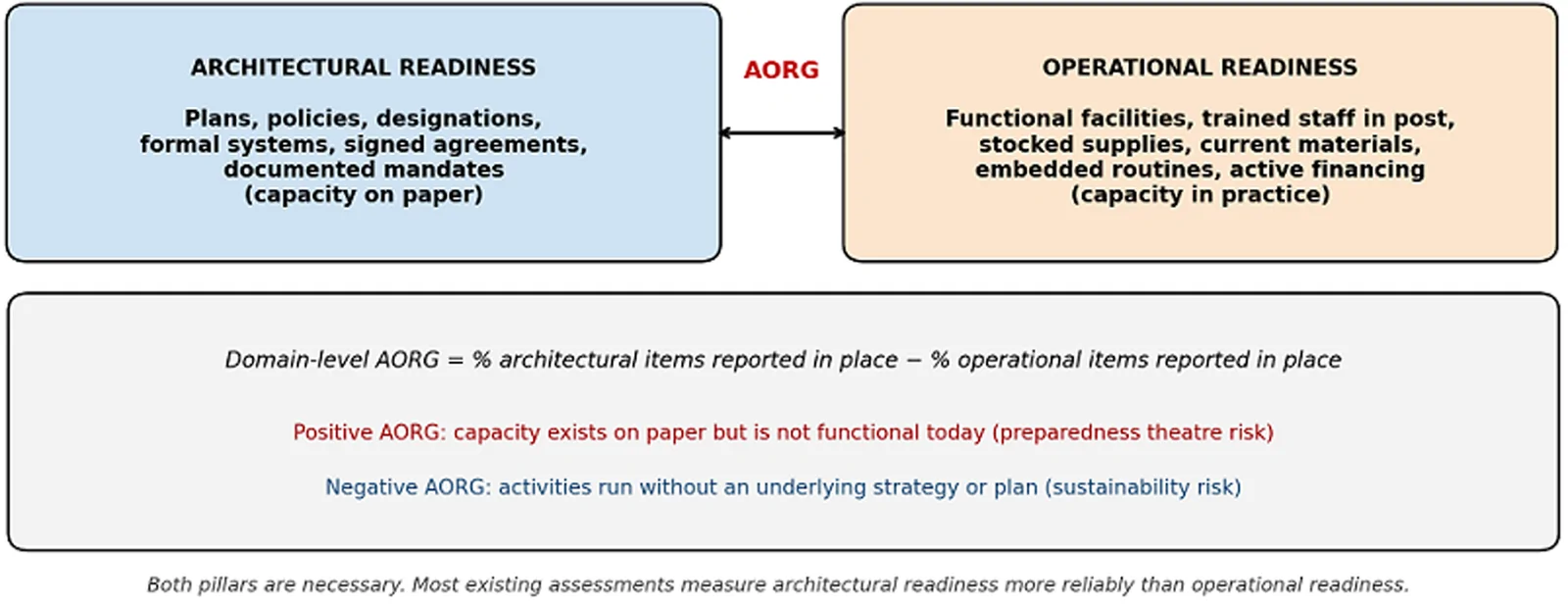
Architectural and Operational Readiness Gap (AORG) framework

This study had three pre-specified objectives. First, to describe Sierra Leone's reported Ebola and filovirus preparedness during the May 2026 BVD PHEIC, using the RCSDC rapid assessment questionnaire as the data source. Second, to develop and apply the AORG framework to the completed questionnaire, distinguishing reported architectural readiness from reported operational readiness at domain and national levels. Third, to integrate the quantitative AORG findings with rapid qualitative content analysis of the seven open-text responses, to identify actionable time-bound operational priorities. No formal hypotheses were pre-specified; the AORG framework was applied as an exploratory analytic lens rather than as a tested measure.

## Methods

**Design:** we used a cross-sectional, convergent parallel mixed-methods design [18]. The study is cross-sectional because data on Sierra Leone's reported Ebola and filovirus preparedness were generated at a single point in time, in May 2026, with no follow-up. The quantitative and qualitative strands were generated from the same instrument at the same time. We analysed them independently and integrated them at the interpretation phase. Reporting follows the Strengthening the Reporting of Observational Studies in Epidemiology (STROBE) cross-sectional checklist as the primary reporting guideline, together with the Good Reporting of A Mixed Methods Study (GRAMMS) [19] and the Standards for Reporting Qualitative Research (SRQR) [20].

**Setting:** Sierra Leone is a West African country with an estimated population of 8.6 million. It shares land borders with Guinea and Liberia and is served by Lungi International Airport. The NPHA was established under the Public Health Act, 2022 (Act No. 17 of 2023) and launched on 14 December 2023. The Agency coordinates national preparedness, surveillance, laboratory and emergency response functions under the Ministry of Health. The NPHA serves as the IHR National Focal Point [10,11].

**Instrument:**the RCSDC Rapid Assessment Questionnaire for Ebola and Filovirus Preparedness Capacity in ECOWAS Member States is a bilingual online instrument (English and French). The RCSDC distributed it to Member States after the 17^th^ May 2026 PHEIC statement [21]. The instrument has three components. First, 35 binary (Yes/No) indicators covering seven operational domains: coordination and governance; points of entry; surveillance and early warning; laboratory; isolation and case management; infection prevention and control; risk communication and community engagement. Second, two categorical multiple-select items on laboratory testing modalities and species coverage. Third, seven open-text fields on perceived challenges and urgent needs.

**Unit of analysis, eligibility and study size:** the unit of analysis was the single completed institutional self-assessment, with the 35 binary indicators within it as the items scored for the AORG calculation. No individual-level participants were recruited and no sampling was performed at the level of individuals or facilities. The study size was therefore fully determined by the structure of the RCSDC instrument: a complete enumeration of all 35 binary indicators, two categorical laboratory items and seven open-text responses, with no items excluded. The item-level enumeration and classification are provided in Annex 1. The unit eligibility criterion was completion of the RCSDC questionnaire by the NPHA Emergency Preparedness and Response team during the operational window of the May 2026 BVD PHEIC; only the completed Sierra Leone return was eligible for this analysis.

**Data collection:** in May 2026, the Emergency Preparedness and Response (EPR) team at the NPHA completed the questionnaire, led by the manager. The team consulted technical leads in surveillance, laboratory, points of entry, infection prevention and control, and risk communication units. No stock, training or facility records were independently verified. The NPHA Executive Director authorised use of the completed instrument and granted permission to publish derived findings. The RCSDC granted permission to use the questionnaire. No responses were missing or partial.

### Quantitative strand

Two members of the study team independently classified each of the 35 binary indicators as architectural or operational. We used a pre-specified rule set. Architectural indicators described the existence of a plan, policy, designated facility, formal system, signed agreement or documented mandate. Operational indicators described functional, equipped, trained, current, disseminated, stocked or active capacity. We resolved initial discrepancies by consensus. The full item-level classification, with response, score and rationale for each indicator, is provided as Annex 1. We calculated domain-level architectural and operational readiness scores as the percentage of items reported as in place within each category. AORG was the percentage-point difference between the two scores. We computed national-level AORG across all 35 binary indicators. Denominator sizes vary across domains. We report all scores with n/N. We frame AORG values as exploratory rather than as estimates of true national readiness. The two categorical laboratory items sit outside the binary AORG calculation. We report the species-coverage finding separately as a critical non-binary observation.

Statistical analysis was descriptive. Because the dataset is a complete enumeration of all 35 binary indicators from a single institutional respondent rather than a sample from a defined population, we did not compute confidence intervals, p-values or other inferential statistics, and no confounder-adjusted estimates were produced. No subgroup or interaction analyses were conducted beyond stratification by domain. No responses were missing or partial, so no imputation or missing-data procedures were required. We did not undertake formal sensitivity analyses; however, we report all domain scores with their full n/N denominators (Table 1) so that readers can identify domains in which the AORG estimate rests on a small architectural or operational denominator and should be interpreted with caution. Quantitative variables were handled in their original binary form and were not categorised or transformed.

**Table 1:** reported architectural readiness, operational readiness and exploratory AORG index by domain, with denominators

Domain	Architectural (n/N, %)	Operational (n/N, %)	AORG (pp)	Notable feature
Coordination & governance	2/2 (100%)	2/3 (67%)	+33	Trust fund exists architecturally; operational triggers for rapid mobilisation not reported as in place
Points of entry & border health	2/2 (100%)	0/1 (0%)	+100*	PoE staff not trained; no holding unit (single operational item)
Surveillance & early warning	3/3 (100%)	3/3 (100%)	0	Listed components reported as in place; field performance not measured
Laboratory capacity	3/3 (100%)	3/4 (75%)	+25	Binary score does not capture species-coverage finding (Zaire only)
Isolation & case management	1/3 (33%)	0/1 (0%)	+33*	Units present but not functional; no isolation at PoE
Infection prevention & control	1/1 (100%)	0/5 (0%)	+100*	Guidelines exist; no operational IPC components reported as in place
Risk communication & engagement	0/1 (0%)	2/3 (67%)	−67*	Activities run without a current Ebola-specific strategy
National total	12/15 (80%)	10/20 (50%)	+30	

* Domain-level AORG rests on a small operational or architectural denominator (≤ 1 item). pp, percentage points. AORG = % architectural items reported in place − % operational items reported in place. AORG = % architectural items reported in place − % operational items reported in place. AORG: Architectural and Operational Readiness Gap, PoE: Power over Ethernet, IPC: Infection Prevention and Control

**Qualitative strand:**we extracted the seven open-text responses verbatim. Given the size of the dataset, we used rapid qualitative content analysis [22] rather than full reflexive thematic analysis. Two team members independently coded the responses inductively. Codes were clustered into candidate themes through discussion. We retained themes that cut across more than one operational domain. Verbatim extracts accompany each theme.

**Integration:** we integrated the two strands through a side-by-side joint display (Table 2) [23]. Each quantitative finding at the domain level was paired with the most relevant qualitative themes. Meta-inferences examined whether qualitative themes explained, extended or contradicted the quantitative findings. We applied the AORG framework post hoc to the completed assessment.

**Table 2:** joint display of quantitative findings (with denominators) and qualitative themes by domain

Domain	AORG (pp) and denominators	Dominant qualitative themes	Meta-inference
Coordination & governance	+33 (A 2/2, O 2/3)	Supplies/financing (trust fund operationalisation)	Architecture in place; reported operational financing trigger is the binding constraint despite existence of PHETF
Points of entry & border	+100 (A 2/2, O 0/1)	Workforce training; non-functional infrastructure	Single operational item; reported plans without trained staff or physical receiving capacity
Surveillance & early warning	0 (A 3/3, O 3/3)	Workforce training; real-time digital data; decentralisation	Listed components reported as in place; field performance, speed and decentralisation not captured by binary scoring
Laboratory capacity	+25 (A 3/3, O 3/4)*	Supplies (reagents, kits, PPE); workforce training	Binary score does not capture the categorical Bundibugyo diagnostic gap, which is the most urgent operational priority
Isolation & case management	+33 (A 1/3, O 0/1)	Non-functional inherited infrastructure; workforce training; real-time data	Designated infrastructure exists but is not functional; rehabilitation rather than new construction
Infection prevention & control	+100 (A 1/1, O 0/5)	Workforce training; supplies; decentralised production	Largest operational gap by indicator count; guidelines exist but no operational components reported as in place
Risk communication & engagement	−67 (A 0/1, O 2/3)	Decentralised, local-language community preparedness	Operational activity reported without a current Ebola-specific strategy; sustainability risk

* The categorical species-coverage finding (Zaire ebolavirus only) sits outside the AORG calculation but is the most operationally salient laboratory result. A: architectural; O: operational; PHETF: Public Health Emergency Trust Fund; AORG: Architectural and Operational Readiness Gap.

### Bias and reflexivity

Three principal sources of bias were anticipated. First, social desirability and institutional self-presentation bias: the questionnaire was completed by the institution responsible for the function under assessment, with no external verification of stocks, training records, isolation infrastructure or financial provisions. Second, measurement bias arising from the binary structure of the instrument, which forces a Yes/No response and cannot capture partial readiness or degree of operational functionality. Third, classification bias in the post hoc allocation of indicators to architectural and operational categories, where instrument wording was ambiguous (notably items C2, F1, F3, F4 and H4 in Annex 1). We addressed these by: explicit framing of all results as reported rather than independently measured; verbatim extraction of all open-text responses; independent dual classification of indicators by two reviewers with consensus resolution and full disclosure of borderline items in Annex 1; and explicit acknowledgement of the small operational or architectural denominators in three domains (points of entry, infection prevention and control, and risk communication). The corresponding author is a physician-researcher and Strategic Advisor at the NPHA. The senior author leads the Agency. Several authors hold dual positions as researchers and as participants in the system under assessment. We address the resulting risks through transparent methods, verbatim qualitative extracts, explicit reframing of all results as reported rather than independently measured, and external peer review through journal submission.

**Ethical considerations:** this is a secondary analysis of an institutional self-assessment carried out under the regulatory mandate of the NPHA, in support of RCSDC-led regional preparedness coordination. No individual-level data were collected. A written institutional determination from the NPHA Executive Director stating that formal ethical review was not required, and granting permission to publish, accompanies this submission. RCSDC permission to use the questionnaire and publish derived findings also accompanies the submission.

## Results

The completed institutional self-assessment yielded responses to 35 binary indicators across seven operational domains, two categorical laboratory items, and seven open-text narrative responses. No responses were missing or partial. Item-level enumeration, classification (architectural or operational), response, score and rationale for all 35 binary indicators are provided in Annex 1. Domain-level reported architectural and operational readiness scores, with denominators, and the corresponding exploratory AORG index, are summarised in Table 1and visualised in Figure 2. The integrated joint display of quantitative and qualitative findings by domain is provided in Table 2.

**Figure 2:**
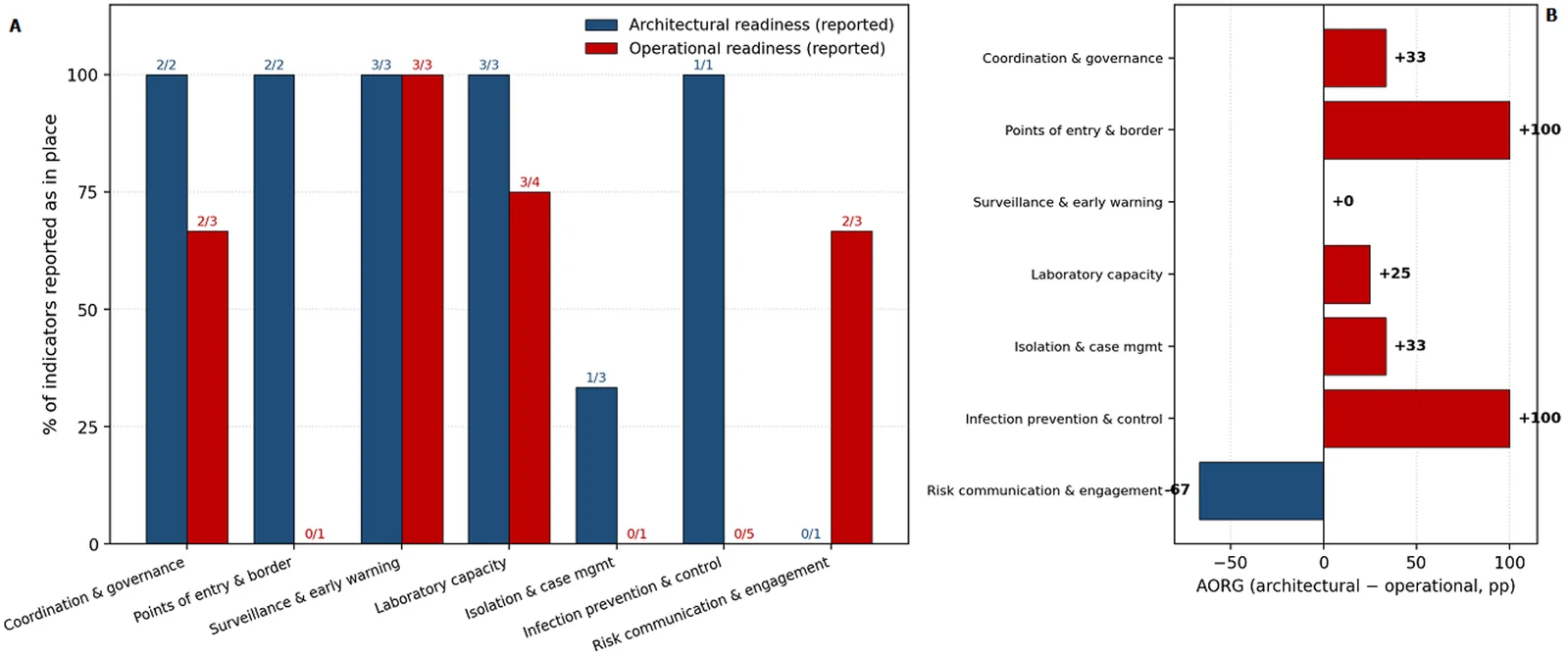
reported architectural and operational readiness for Ebola and filovirus disease in Sierra Leone, May 2026 institutional self-assessment, with denominators; A) percentage of indicators within each category reported as in place; n/N values shown above each bar; B) exploratory AORG index by domain

### Quantitative findings

#### Reported readiness by domain

Coordination and governance. The NPHA reported a national preparedness and response plan for viral haemorrhagic fevers, and a separate Ebola and filovirus plan (developed in 2021, currently under review). The plan was reported as operational. A national One Health coordination platform was reported as active. No financial provisions were reported as in place for rapid mobilisation of emergency funds, despite the existence of the Public Health Emergency Trust Fund (PHETF) established alongside the NPHA in December 2023 [12]. Points of entry and border health. Standard operating procedures for handling ill travellers and referral mechanisms for suspected cases at borders were reported as in place. Point of entry staff were not reported as fully trained on Ebola and filovirus preparedness.

Surveillance and early warning: the listed components (event-based surveillance, indicator-based surveillance, dissemination of case definitions, community-based surveillance, a 24/7 alert hotline, and cross-border surveillance) were all reported as in place. These findings reflect the reported presence of the listed components and do not measure alert sensitivity, timeliness, completeness or field performance.

Laboratory capacity: National laboratory testing capacity was reported as in place, with six laboratories described as capable of testing. Modalities reported as available were polymerase chain reaction (PCR), antigen detection, antibody-based rapid diagnostic test, and sequencing. Species coverage was reported as limited to Zaire ebolavirus; Sudan, Bundibugyo and other filovirus species were not reported as covered. Laboratory personnel were reported as trained for Ebola testing and in biosafety and biosecurity. Safe specimen collection, an established specimen referral system and agreements with regional or international reference laboratories were all reported as in place. Personal protective equipment for laboratory staff was reported as insufficient.

Isolation and case management: isolation units were reported as available at healthcare facilities but as not currently functional or adequately equipped. No isolation capacity was reported at the international airport or at land borders.

Infection prevention and control: national IPC guidelines were reported as in place. None of the listed operational components (healthcare worker training at all levels, availability of guidelines and SOPs at facilities, PPE availability at all levels, healthcare-associated infection monitoring, waste management adequate for an Ebola response) were reported as in place. Risk communication and community engagement. Rumour tracking, social listening and media engagement mechanisms were reported as in place. The country was reported as not currently having a national risk communication and community engagement strategy specific to Ebola preparedness. Communication materials were not reported as available in local languages.

### Exploratory architectural and operational readiness scores

Across the 35 binary indicators, 15 were classified as architectural and 20 as operational (Annex 1). Architectural readiness was reported as in place for 12 of 15 items (80 percent); operational readiness for 10 of 20 items (50 percent). The exploratory national AORG was +30 percentage points. The pattern by domain is shown in Figure 2 (Panel A for reported readiness and Panel B for the AORG index) and summarised in Table 1. Denominator sizes vary substantially: the +100 percentage-point AORGs for points of entry and for IPC rest on small operational denominators (1 and 5 items respectively), and the -67 percentage-point AORG for risk communication rests on a single architectural item. These domain-level scores should be read as indicative rather than as ranked estimates.

#### Critical non-binary finding: species-specific diagnostic coverage

Sierra Leone reported national capacity in PCR, antigen detection, antibody-based rapid diagnostic testing and sequencing across six laboratories. Reported species coverage was limited to Zaire ebolavirus. Sudan ebolavirus, Bundibugyo ebolavirus and other filovirus species were not reported as covered. This categorical finding is not captured in the binary AORG score. It is of high operational salience in the current PHEIC, which is caused by Bundibugyo virus [1,2]. The binary AORG score for the laboratory domain (+25 pp) therefore understates the seriousness of the diagnostic gap.

### Qualitative findings

Rapid qualitative content analysis of the seven open-text responses generated four cross-cutting themes.

**Theme 1: workforce training and refresher needs:** training featured in every domain-specific response. Reported needs spanned several cadres (point of entry staff, surveillance officers, chiefdom supervisors, healthcare workers, laboratory staff, call centre and watch staff, community health workers and community animal health workers) and content areas (case detection, sample collection, biosafety, IPC, real-time reporting). The explicit mention of refresher training indicated that initial training had been delivered in some areas but had not been sustained.

**Theme 2: supplies, personal protective equipment, reagents and supply-chain fragility:** reagents for non-Zaire species, sequencing reagents, sample collection kits including blood tubes and viral transport media, laboratory-specific PPE, IPC supplies, cleaning materials, soap and alcohol-based hand sanitisers were all reported as urgent needs. The response to supply-chain fragility was framed in one instance in terms of localised production rather than only external procurement.

**Theme 3: non-functional inherited infrastructure and real-time data systems:** two sub-strands appeared. The first concerned physical infrastructure inherited from earlier emergency response but not currently functional, framed in the language of rehabilitation. The second concerned electronic tools for real-time reporting (tablets, computers, internet modems, real-time mortality surveillance), repeated across surveillance, isolation and case management domains.

**Theme 4: decentralised, local-language community preparedness:** the responses repeatedly located readiness at sub-national and community level. Chiefdom supervisors, community-based surveillance, community health workers and community animal health workers, alongside traditional healers, religious leaders, influencers and persons with disabilities, were named as priority targets for orientation and engagement. Materials were framed as requiring translation and local production.

### Integration

Table 2 presents a joint display of the quantitative AORG findings and qualitative themes by domain. Where the quantitative finding showed a positive AORG, the qualitative themes consistently identified the operational ingredients reported as absent. These were most often training, supplies and functional infrastructure. The inverse pattern in risk communication (operational activities running without a current strategy) was paired with qualitative emphasis on localisation, translated materials and stakeholder orientation. This suggests that the operational substrate exists but requires strategic codification.

## Discussion

In this single institutional self-assessment, the NPHA reported 12 of 15 architectural indicators and 10 of 20 operational indicators on the RCSDC instrument as in place. The exploratory national AORG was +30 percentage points. The gap concentrated in operational shortfalls in IPC, isolation, points of entry, and the categorical laboratory finding on Bundibugyo virus diagnostic capacity. Surveillance was the only domain where architectural and operational components were both reported as fully in place. Risk communication and engagement showed an inverse pattern. Operational activities ran in the absence of a current Ebola-specific strategy. Four cross-cutting qualitative themes explained or extended the quantitative findings: workforce training; supplies and supply-chain fragility; non-functional inherited infrastructure; and decentralised local-language community preparedness. We propose AORG as an exploratory analytic lens, not a validated preparedness index. It complements existing instruments. The Joint External Evaluation, the States Parties Annual Reporting tool and the Universal Health and Preparedness Review already encode progression from formal to operational capacity to varying degrees [13-15]. The contribution of AORG is to offer a transparent, rapid analytic step. The step can be applied to existing structured assessments under time pressure. It can be paired with rapid qualitative analysis of accompanying open-text data. The framework depends on the underlying instrument, the accuracy of self-report, the indicator set, the item classification, and the absence of external verification. Domain scores resting on small denominators should be read as indicative.

The most operationally consequential single finding is the reported limitation of national diagnostic capacity to Zaire ebolavirus. The current PHEIC is caused by Bundibugyo virus. Six testing laboratories, trained personnel, safe specimen handling and an established referral system are substantial assets. These assets cannot deliver species identification for the current outbreak strain without external reference laboratory support. The implication is delay at the very phase of an outbreak where confirmation speed is decisive. The same operational pattern was observed at the start of the current DRC outbreak. Initial samples from Bunia tested negative on Zaire-calibrated equipment before being confirmed in Kinshasa as Bundibugyo virus [3]. The highest-priority operational investment surfaced by this assessment is procurement of Bundibugyo-compatible PCR reagents, viral transport media and sample collection kits, with confirmed referral pathways to regional or international reference laboratories [24].

The IPC domain showed the largest operational gap by indicator count: zero of five operational items reported as in place. This matters for healthcare worker safety. A substantial proportion of Sierra Leonean healthcare workers were infected during the 2014 to 2016 epidemic [25]. National guidelines exist. Reported training, supplies and facility-level dissemination do not. The pattern is consistent with the wider observation that outbreak-era investments decay during inter-epidemic periods [26]. The qualitative emphasis on local production of soap and alcohol-based hand sanitisers aligns with the African Union New Public Health Order. Expanded local manufacturing of public health commodities is one of its pillars [27]. The inverse AORG in risk communication and engagement is methodologically and substantively informative. Operational activities (rumour tracking, social listening, media engagement) were reported as in place. A current Ebola-specific strategy was not. The 2014 to 2016 epidemic in Sierra Leone showed that community engagement, including safe and dignified burials, was decisive in ending transmission. The current self-assessment suggests the operational substrate persists. Strategic codification has not been refreshed since 2021. The qualitative theme of decentralised, local-language community preparedness identifies what a refreshed strategy should integrate: translated materials, structured engagement of traditional and religious leaders, and inclusion of community health workers, community animal health workers and persons with disabilities.

The reported absence of operational financial provisions is notable given the existence of the PHETF. The Trust Fund was established alongside the NPHA in December 2023 [12]. The architecture is in place. The operational pathway for rapid mobilisation, including triggers, capitalisation and disbursement mechanisms, was not reported as functional at the time of assessment. This is a tractable institutional reform. It would substantially shorten the operational distance between architecture and practice during a public health emergency. These findings, from a single institutional self-assessment, do not on their own justify changes to global instruments. They suggest that future preparedness measurement could benefit from more explicit distinction between formal capacity and operational functionality. This applies under the amended IHR (2005) and under the WHO Pandemic Agreement adopted in May 2025 [4,16]. We frame this as an exploratory implication for further work.

### Implications for Sierra Leone and the West African region

Sierra Leone's findings have implications beyond one country. Many African countries built similar formal preparedness architecture after the 2014 to 2016 epidemic. National plans were drafted. Public health agencies were established. Trust funds were created. The current PHEIC tests whether that architecture functions. Three regional implications stand out. First, on diagnostics. Many ECOWAS laboratories are calibrated for Zaire ebolavirus alone. The current PHEIC is caused by Bundibugyo virus. The DRC pattern, in which initial Zaire-calibrated samples were negative before Bundibugyo confirmation in Kinshasa [3], could be repeated elsewhere. ECOWAS Member States should verify Bundibugyo PCR coverage at their reference laboratories. Referral pathways should be confirmed before the first suspected case arrives. Second, on IPC. The decay of operational IPC during inter-epidemic periods is unlikely to be unique to Sierra Leone. Regional coordinating bodies, including WAHO and Africa CDC, could support periodic facility-level IPC readiness verification. Local production of IPC commodities, in line with the African Union New Public Health Order [27], could reduce supply-chain fragility across the region. Third, on emergency financing. Many African countries established public health emergency funds after the 2014 to 2016 epidemic. Architectural establishment does not guarantee operational mobilisation. ECOWAS Member States and Africa CDC could promote regional benchmarking of trust fund operationalisation, including triggers, capitalisation thresholds and disbursement pathways. The AORG framework, applied to the same RCSDC instrument across other ECOWAS Member States, would support direct regional comparison. A regional AORG dataset would inform both country-level investment and ECOWAS coordination during the current and future emergencies.

### Strengths and limitations

This study has several strengths. The assessment was completed by the institutional team with operational responsibility for the function under study. The mixed-methods design paired the structured quantitative architecture of the RCSDC instrument with verbatim open-text data on perceived needs. The exploratory AORG framework offers a transparent, replicable analytic step. The study is reported in accordance with the STROBE cross-sectional checklist, with the mixed-methods elements additionally reported per GRAMMS and the qualitative strand per SRQR.

Generalisability is bounded. The quantitative AORG values are specific to Sierra Leone in May 2026 and to the RCSDC instrument; they are not generalisable as national readiness estimates for other countries, time points or instruments. The AORG framework itself, however, is portable: any structured rapid preparedness assessment that distinguishes documented capacity from functional capacity can be analysed using the same architectural/operational classification and the percentage-point gap calculation. The findings on diagnostic species coverage, decay of operational IPC between outbreaks and the gap between architectural and operational status of national emergency financing instruments are plausibly relevant to other ECOWAS Member States and to other African countries that built similar formal preparedness architecture after the 2014 to 2016 epidemic; empirical confirmation in those settings remains to be done.

Important limitations should be acknowledged. The dataset reflects the institutional position of a single national public health agency at one point in time. Binary response options do not capture partial readiness. No independent facility verification was conducted. The instrument was designed for rapid operational assessment, not formal research, and has not been independently validated. The AORG classification was applied post hoc and depends on the indicator set in the instrument. Several domain scores rest on small denominators. The qualitative dataset is short and institutionally generated. We have used the modest descriptor of rapid qualitative content analysis. Several authors hold roles within the agency under assessment. The implications are addressed in the reflexivity statement and the competing interests declaration. Findings should not be interpreted as an independent national audit. Future work could triangulate this self-assessment with the most recent Joint External Evaluation, after-action review reports, simulation exercises, and key-informant interviews with the frontline cadres named in the analysis.

**Recommendations:** time-bound priorities arising from the integrated findings, with named responsible institutional units.

#### Immediate (0 to 30 days)

The NPHA Laboratory Directorate, with regional and international reference laboratory partners, should procure Bundibugyo-compatible PCR reagents, sequencing reagents and sample collection materials, and confirm referral pathways. The NPHA Emergency Preparedness and Response Directorate, with the Directorate of Hospital and Laboratory Services, should rehabilitate priority isolation units and pre-position PPE and IPC supplies at high-risk facilities. The NPHA Workforce Development and Training Unit, with the National Disease Surveillance Programme and Port Health Unit, should deliver targeted training for points of entry, IPC, laboratory, surveillance and call-centre staff. The NPHA Risk Communication and Community Engagement Unit, with district health management teams, should produce materials in priority local languages and orient traditional and religious leaders, influencers and disability advocacy groups.

#### Short-term (1 to 3 months)

The NPHA Emergency Preparedness and Response Directorate should finalise the ongoing update of the national Ebola and filovirus preparedness and response plan. The NPHA RCCE Unit should finalise and disseminate an Ebola-specific risk communication and community engagement strategy. The Port Health Unit, with the Sierra Leone Civil Aviation Authority and border districts, should establish holding capacity at Lungi International Airport and priority land borders. The National Disease Surveillance Programme should strengthen real-time mortality and alert reporting through electronic tools and training of chiefdom supervisors and call-centre staff. The NPHA should conduct a national simulation exercise.

#### Medium-term (3 to 12 months)

The Ministry of Health, with the Ministry of Finance and the NPHA Board, should define and operationalise epidemiological triggers and disbursement pathways for the PHETF. The NPHA Workforce Development and Training Unit should institutionalise refresher training cycles. The Directorate of Hospital and Laboratory Services should maintain isolation units between outbreaks through routine budget lines. The Ministry of Trade and Industry, with NPHA and the National Pharmaceutical Procurement Unit, should develop local production capacity for IPC commodities. The NPHA Research and Evaluation Unit should repeat the AORG assessment at defined intervals.

## Conclusion

During the May 2026 BVD PHEIC, the NPHA reported substantial formal preparedness architecture but a notable operational readiness gap. The gap concentrated in Bundibugyo-specific diagnostics, infection prevention and control, isolation infrastructure, risk communication strategy, and operationalisation of the existing emergency financing trust fund. The exploratory AORG framework surfaces this gap transparently. The findings reflect the reported institutional position of one agency at one point in time. They are not an independent national audit. They offer a tractable, time-bound set of operational priorities for Sierra Leone, and a usable analytic step for rapid mixed-methods preparedness measurement across the West African region and other settings.

### What is known about this topic

- The World Health Organization (WHO) declared the Bundibugyo Ebola virus disease (EVD) outbreak in the Democratic Republic of the Congo (DRC) and Uganda a Public Health Emergency of International Concern (PHEIC) on May 17, 2026; the event occurs in the absence of any licensed vaccine or specific therapeutic for Bundibugyo virus, with past outbreak case-fatality rates of 30 to 50 percent;
- Existing rapid preparedness assessment instruments and country self-assessments under the International Health Regulations tend to capture the presence of plans, designated facilities and formal systems more reliably than their current operational functionality, particularly during the early hours and days of an active public health emergency;
- Sierra Leone's 2014 to 2016 Ebola epidemic experience drove a decade of institutional reform, including enactment of the Public Health Act, 2022 (Act No. 17 of 2023) and establishment of the National Public Health Agency and Public Health Emergency Trust Fund in December 2023.

### What this study adds

- A transparent, replicable analytic lens, the Architectural and Operational Readiness Gap (AORG) framework, for distinguishing formal preparedness architecture from reported operational functionality in rapid mixed-methods assessments during public health emergencies;
- Empirical application in Sierra Leone during the May 2026 BVD PHEIC, showing a +30 percentage-point national AORG and concentrated operational gaps in Bundibugyo diagnostics, infection prevention and control, isolation infrastructure and the operational status of the existing emergency financing trust fund;
- A time-bound set of operational priorities for Sierra Leone (0 to 30 days, 1 to 3 months, and 3 to 12 months), each with a named responsible institutional unit, providing an immediately usable accountability framework for national preparedness investment.

## Annexes

**Annex 1**: supplementary material
